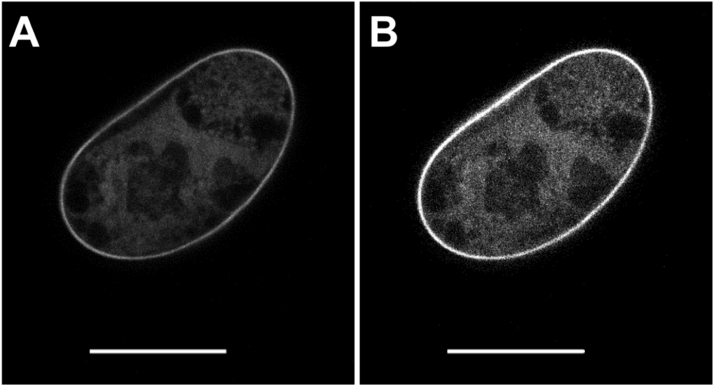# Electropermeabilization-based fluorescence *in situ* hybridization of whole-mount plant parasitic nematode specimens

**DOI:** 10.1016/j.mex.2019.11.009

**Published:** 2019-11-13

**Authors:** Casey L. Ruark-Seward, Eric L. Davis, Tim L. Sit

**Affiliations:** Department of Entomology and Plant Pathology, North Carolina State University, Raleigh, NC, USA

**Keywords:** Fluorescence *in situ* hybridization (FISH), Nematode, RNA, FISH, Localization, Fluorescence, Gene expression, Virus

## Abstract

A fluorescence *in situ* hybridization (FISH) protocol was developed for nematodes in which nucleic acid probes are introduced within the organism *via* electroporation. This modification of existing FISH protocols removes numerous chemical wash steps, and thus, reduces protocol time and specimen loss while improving hybridization sensitivity. The presented work is optimized for juveniles of soybean cyst nematode (SCN; *Heterodera glycines*) and has been used to identify both host and associated-microbial (viral) targets. Moreover, through the use of two different long wavelength fluorophores, two probes can be colocalized within one individual. This protocol may be adapted to identify targets-of-interest within other life stages and nematode species.

This protocol improves:

• Hands-on protocol time (by approximately 1.5 h).
• Specimen loss (fewer aspiration steps).
• Hybridization (allows colocalization with two nucleic acid probes and increases sensitivity).

## Graphical abstract

**Figure:**
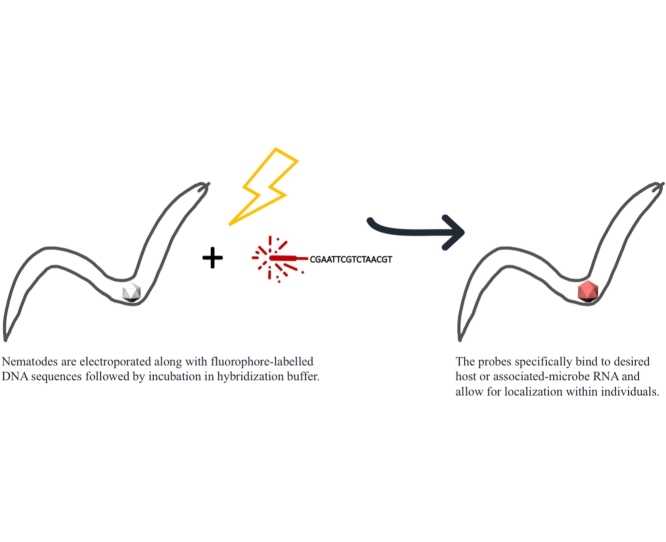

**Specification Table**

**Table:** 

Subject Area:	Agricultural and Biological Sciences
More specific subject area:	molecular nematology
Method name:	fluorescence *in situ* hybridization (FISH)
Name and reference of original method:	Original FISH protocol: Vandekerckhove, T.T., Coomans, A., Cornelis, K., Baert, P., and Gillis, M. Use of the *Verrucomicrobia*-specific probe EUB338-III and fluorescent *in situ* hybridization for detection of “*Candidatus* Xiphinematobacter” cells in nematode hosts. *Appl. Environ. Microbiol.*, 68 (6): 3121–3125 (2002). Modification which uses same protocol as Vandekerckhove et al. [3] but includes newer fluorophore (Cy5 replaced with ATTO-633): Brown, A.M.V., Howe, D.K., Wasala, S.K., Peetz, A.B., Zasada, I.A., and Denver, D.R. Comparative genomics of a plant-parasitic nematode endosymbiont suggest a role in nutritional symbiosis. *Genome Biol. Evol.* 7 (9): 2727–2746 (2015). Electroporation settings: Kines, K.J., Rinaldi, G., Okatcha, T.I., Morales, M.E., Mann, V.H., Tort, J.F., and Brindley, P.J. Electroporation facilitates introduction of reporter transgenes and virions into schistosome eggs. *PLOS Negl. Trop. Dis*. 4 (2): e593 (2010).
Resource availability:	NA

## Method details

Plant parasitic nematodes (PPN) cause major economic damage to food crops, turf, and ornamental plants [1]. The microscopic size and tough cuticle of PPN provide a challenge to traditional molecular localization experiments such as RNA *in situ* hybridizations (ISH). The primary ISH protocol available for PPN localized gene expression involves chemical fixation, segmenting the specimen, and several permeabilization steps in order to gain probe entry into nematode cells and tissues [2]. However, a whole-mount fluorescence *in situ* hybridization (FISH) technique was developed by Vandekerckhove et al. [3] to localize intracellular symbionts within nematodes. Later, this methodology was again utilized within PPN to successfully localize endosymbiotic bacteria [4,5]. The technique used chemical preparation of the cuticle to overcome some of the largest barriers for FISH assays in nematodes: fluorescent nucleic acid probes are introduced in one step and are small enough in size (approximately 6.8 kDa) to pass through the cuticle, and the fluorescent dye utilized has a longer emission wavelength (633 nm) to avoid the intense autofluorescence of nematode tissues observed at shorter wavelengths.

In this report we reduce the complexity of the protocol (Table 1) originally described by Vandekerckhove et al. [3] to detect RNA targets within nematodes while increasing protocol sensitivity. To facilitate probe entry within the nematode, several time-intensive incubation and wash steps were replaced by square-wave electroporation [6]. The revised protocol reduces experiment time by approximately 1.5 h and lowers nematode specimen loss due to reduced aspiration steps. Moreover, the electroporation protocol decreases preparation complexity as it requires a smaller number of chemical reagents. Additionally, this protocol has been adjusted to allow for two RNA targets to be colocalized at the same time (absorbances of 565 nm and 647 nm) when viewed on an appropriate confocal fluorescence microscope.

**Table 1:** **A comparison of fluorescence *in situ* hybridization (FISH) protocols.** The chemical permeabilization steps replaced with electroporation are described. Following these steps, samples from both methods are ready for hybridization buffer. The chemical method will also need the addition of fluorescent nucleic acid probes at this time.

Chemical Permeabilization Wash Steps (old)	Electropermeabilization (new)
1. 0.85 w/v NaCl (2 min)	1. Electroporate nematodes in sterile water with probes (20 ms)
2. 0.85 w/v NaCl (2 min)	
3. 1:1 v/v glacial acetic acid & 100% ethanol (10 min)	
4. 100% ethanol (5 min)	
5. 100% ethanol (5 min)	
6. 1:1 100% methanol & phosphate buffered Tween 20 (PBT) (10 min)	
7. 1% formaldehyde in PBT (30 min)	
8. PBT (2 min)	
9. PBT (2 min)	

The presented FISH electroporation protocol has been optimized for pre-parasitic second-stage juveniles (ppJ2s) of soybean cyst nematode (SCN), a pathogen of major economic concern [7], and it has been successfully used to localize both host mRNA and endosymbiotic viral RNA. It is the authors’ aim that this procedure be useful for those interested in localizing nematode gene expression and microbial endosymbionts, and the method could be expanded to additional RNA targets, life stages, and species.

## Fluorescent probe design

Probes consisted of a 20-mer single-stranded DNA oligo with an ATTO dye (ATTO-TEC; Siegen, Germany) attached to the 5’ end. DNA sequences were selected to bind to the mRNA of nematode genes and the genomic strand of viral targets. Probes were synthesized by Eurofins Genomics (Louisville, KY, USA). To overcome background autofluorescence within SCN ppJ2s, longer wavelength fluorophores were conjugated to DNA probes. Viewing untreated ppJ2s with a laser scanning confocal microscope (Zeiss LSM 880) demonstrated that fluorophores with an absorption value of 565 nm or longer are optimal. As such, two dyes can be utilized for colocalization without laser channel bleed-through: ATTO-565 and ATTO-647N. Dyes below this wavelength can be difficult to distinguish from background autofluorescence from the nematode (Supplemental Fig. 1). It is recommended that putative nucleic acid probe sequences be analyzed *via* NCBI Blast against the host species to reduce the potential for off-target hybridization.

## Materials & equipment

• Confocal microscope (*i.e.* Zeiss LSM 880)
• Electroporator (*i.e.* GenePulser Xcell from Bio-Rad)
• Microcentrifuge
• Hybridization oven or heat block
• Slides and slide covers
• 1.5 ml tubes
• Pipettes/tips
• 0.4 cm cuvettes

## Reagents

• 0.1 % benzalkonium chloride solution
• Sterile water
• Fluorescent DNA probe(s) in TE buffer
• Hybridization buffer [20 mM Tris-HCl, pH 7.4, 0.02 % w/v sodium dodecyl sulfate (SDS), 0.9 M NaCl, 5 mM ethylenediaminetetraacetic acid (EDTA), 60 % v/v formamide]
• Hybridization wash buffer (20 mM Tris-HCl, 0.02 % w/v SDS, 8 mM NaCl, 5 mM EDTA)
• ProLong Diamond Antifade Mountant (with or without DAPI) (Thermo Fisher Scientific)

## Preparation

Prewarm hybridization buffer and hybridization wash buffer at 46 °C and 50 °C, respectively.

## Hybridization protocol

1 Pellet hatched cohorts of ppJ2s in a 1.5 ml microcentrifuge tube for 2 min at low speed (4000 × *g*).

[Note: Approximately 100 ppJ2s per hybridization reaction is typically used. Fewer nematodes can be used but care must be taken when pipetting to avoid sample loss. Some nematode loss will occur naturally because of stickiness to plastics.]

2 Remove excess water from tubes *via* pipette leaving approximately 10 μl of fluid around the nematode pellet.
3 Surface sterilize nematodes by adding 200 μl of 0.1 % benzalkonium chloride solution to the sample [4], incubating for 1 min at room temperature, and then centrifuging for 1 min at 6000 × *g*.

[Note: Benzalkonium chloride also acts as a surfactant and reduces adherence of nematodes during protocol.]

4 Following centrifugation, carefully draw off approximately 200 μl of supernatant and discard.
5 Rinse nematodes with 200 μl of sterile water, pellet for 2 min at 4000 × *g*, and remove excess liquid.
6 Resuspend samples in 50 μl sterile water for each reaction/probe combination (*i.e.* 200 μl water for 4 different probe reactions).
7 Pipette 50 μl of nematode water suspension into an electroporation cuvette (0.4 cm gap distance), and then add 150 μm of each probe [(1.5 μl of 100 μm concentrated probe in TE (10 mM Tris-HCl, 1 mM EDTA, pH 7.4)].
8 Electroporate nematode/probe sample(s) on an electroporator with a single-pulse square wave at 125 V for 20 ms.
9 Add 100 μl of pre-warmed (46 °C) hybridization buffer [20 mM Tris-HCl, pH 7.4, 0.02 % w/v sodium dodecyl sulfate (SDS), 0.9 M NaCl, 5 mM ethylenediaminetetraacetic acid (EDTA), 60 % v/v formamide] to the nematode suspension.
10 Transfer to a 1.5 ml microcentrifuge tube. Incubate 4 h at 46 °C in the dark on a gently rocking platform.

[Note: A hybridization incubator was utilized for these protocols (Model 1000, Robbins Scientific); however, a stationary heat block will generate similar results if a specialized oven is not available. The heat block could also be placed on a platform rocker.]

## Removal of excess probe

1 Following hybridizations, centrifuge samples for 1 min at 6000 × *g* and remove excess liquid.
2 Perform three incubations at 50 °C in 100 μl of pre-warmed hybridization wash buffer (20 mM Tris-HCl, 0.02 % w/v SDS, 8 mM NaCl, 5 mM EDTA) for 20 min each in the dark on a rocking platform at a slightly higher rocking speed than the hybridization step.
3 Centrifuge at 6000 × *g* for 1 min and remove excess liquid from the nematode pellet.
4 Pipette treated nematodes onto a glass microscope slide in a small volume of remaining wash buffer (approximately 5 μl).
5 Let the sample air dry on the slide until only a small film of water surrounds the nematodes. Then quickly (and without introducing bubbles) add 10 μl ProLong Diamond Antifade Mountant (Thermo Fisher Scientific) and seal with a coverslip.

[Note: ProLong is available with or without DAPI stain incorporated.]

6 Dry slides horizontally in the dark at 25 °C for 24 h and then store at 4 °C in the dark. Slides can reliably be viewed for up to a month.

## Viewing slides

Nematodes can be viewed on an appropriate confocal microscope that is capable of separating dye wavelengths without channel bleed-through. For this protocol, a Zeiss LSM 880 confocal microscope with an Apochromat 40x water immersion objective (1.2W Corr FCS M27) was used, and analysis was conducted with Zeiss Zen version 2.3 software.

## Method validation

**Fig. 1:**
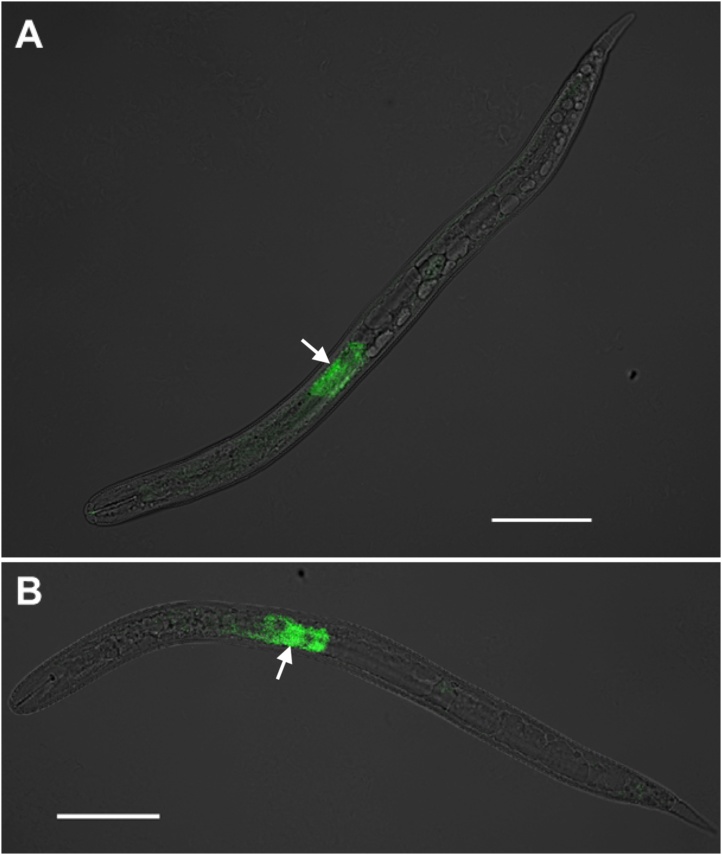
**Comparison of FISH methodologies for fluorescent nucleic acid probe entry into whole nematodes.** Two preparation methodologies for hybridization with fluorescent DNA probes are displayed in soybean cyst nematode (SCN) juveniles. **A.** Results *via* the chemical permeabilization protocol (old); **B.** Results *via* the electropermeabilization protocol (new). Both nematodes were treated with the same ATTO-647N conjugated DNA probe complementary to mRNA of a ubiquitin protein (4G06) that is specifically expressed in the two subventral esophageal gland cells (arrow) in SCN. The brightfield channel is muted but retained in images for better structure clarity. Wavelength color is artificially selected in Zeiss Zen software for optimal image contrast. Scale bar represents 50 μm (Zeiss LSM 880).

**Fig. 2:**
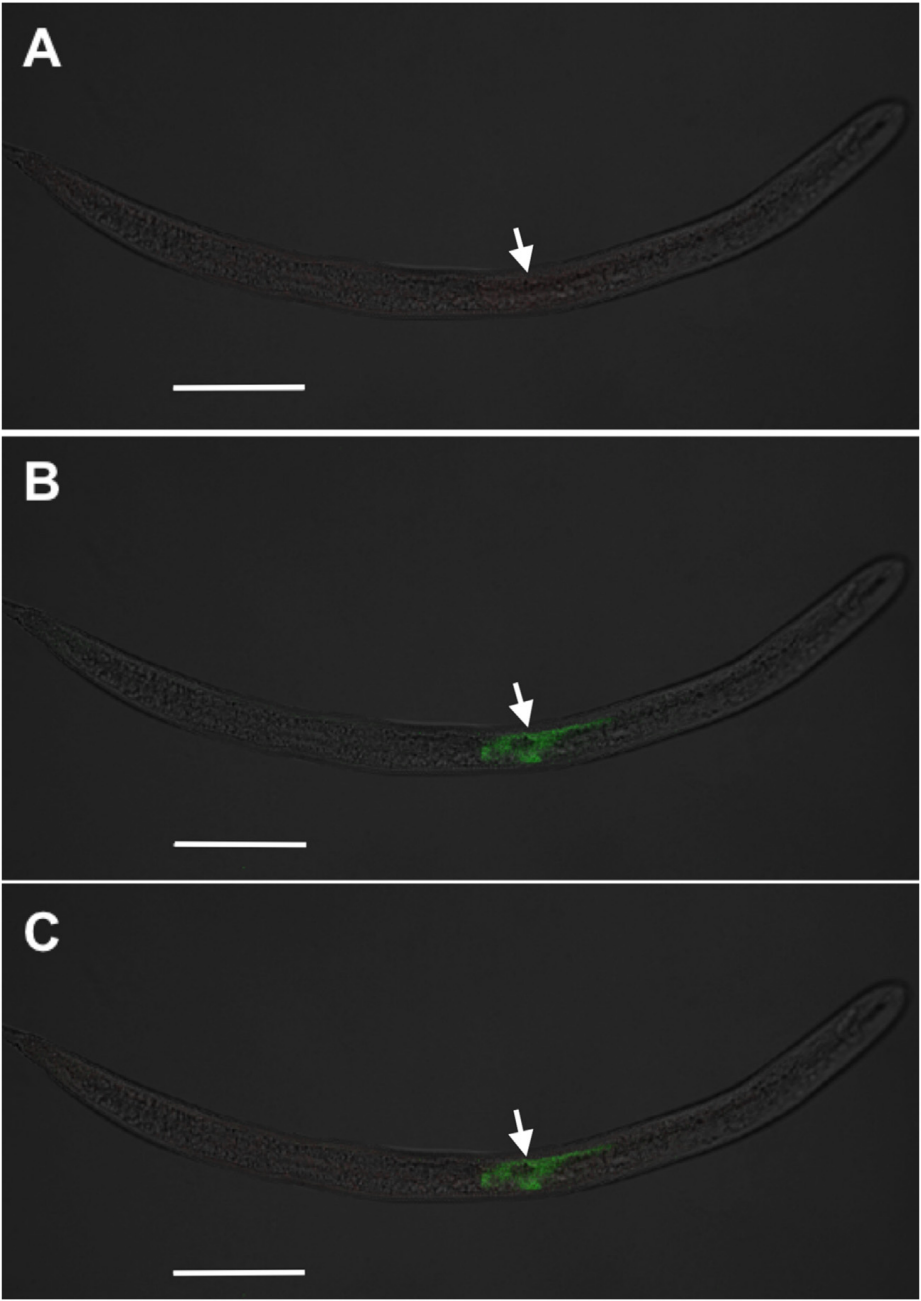
**Localization of non-binding and host subventral gland (SvG) RNA within juveniles of the soybean cyst nematode (SCN)**. **A**: Non-binding (negative control) *Flock house virus* probe single channel [ATTO-565; red], **B**: SvG 4G06 probe single channel [ATTO-647N; green], **C**: overlay. Arrows denote location of SvG. The brightfield channel is muted but retained in images for better structure clarity. Wavelength colors are artificially selected in Zeiss Zen software for optimal image contrast. Scale bar represents 50 μm (Zeiss LSM 880).

**Fig. 3:**
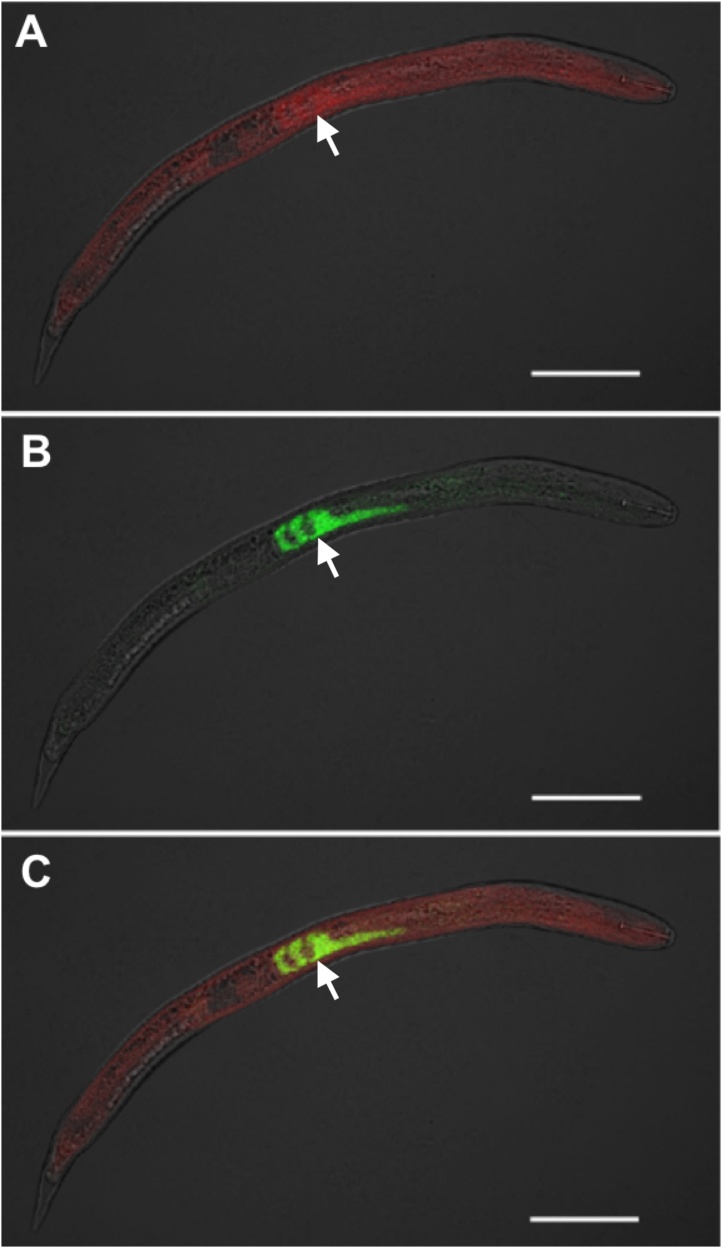
**Colocalization of SCN Bunya-like virus (BLV) RNA and mRNA of SCN subventral gland (SvG) secretory ubiquitin protein (4G06) within nematode juveniles. A**: SCN BLV probe single channel [ATTO-565; red], **B**: SvG (4G06) probe single channel [ATTO-647N; green], **C**: overlay. Arrows denote location of SvG. The brightfield channel is muted but retained in images for better structure clarity. Wavelength colors are artificially selected in Zeiss Zen software for optimal image contrast. Scale bar represents 50 μm (Zeiss LSM 880).

The protocol described by Vandekerckhove et al. [3] and later Brown et al. [4] was adjusted *via* an alternative permeabilization protocol in which nematodes are electroporated with nucleic acid probes. A comparison of SCN ppJ2s labelled *via* the two different permeabilization protocol options can be seen in Fig. 1. These images are a comparison of the same probe specific to a SCN (4G06) secretory ubiquitin protein mRNA [8] that localized within the subventral esophageal gland cells. Both methodologies efficiently hybridized specifically to the target nematode mRNA; however, the electropermeabilization protocol resulted in slightly higher intensity labelling of the gland cells. To determine the stringency of the presented electropermeabilization-based FISH, a non-binding probe was designed to *Flock house virus* (FHV) which does not naturally infect nematodes. A 20-mer FHV DNA sequence was selected that should not bind to RNA of the nematode or any known associated-microbes. When compared with localization of the host (4G06) SvG probe (Fig. 2), the visual absence of the non-binding (FHV) fluorophore suggests this technique is specific to its intended targets. Finally, a naturally infecting virus of SCN, Bunya-like virus (BLV) [9], is demonstrated within metabolically active cells (particularly the SvG) and shown along with the host SvG probe (Fig. 3). Thus, both nematode and microbial endosymbiont nucleic acid targets can be localized within whole nematode specimens with specificity and increased sensitivity, and up to two different fluorophores can be colocalized with this procedure.

## Additional information

Attempts were made to introduce labeled nucleic acid probes into SCN eggs, but it was discovered that eggshells autofluoresced at both 565 nm and 647 nm in addition to the shorter wavelengths seen in juveniles (Supplemental Fig. 2). Additional attempts to permeabilize the vitelline membrane and chitinous layer of eggshells were conducted in an effort to allow probes to penetrate the egg and be visible above background noise at longer wavelengths. Thus, eggs were washed with sodium hypochlorite followed by incubation with chitinase (isolated from *Streptomyces griseus*). Increasing the sodium hypochlorite concentration and wash times dissolved eggshells but resulted in more severe damage to cells and increased autofluorescence at 565 nm above that of untreated samples. Additionally, chitinase treatment had no visible effect without the addition of sodium hypochlorite (tested up to 1.5 h), likely because either the vitelline layer must first be dissolved or this particular type of chitinase from *Streptomyces griseus* was not as effective against SCN chitin compared to *C. elegans* [10].

## Declaration of Competing Interest

The authors declare that there are no conflicts of interest.

## Figures and Tables

**Figure:**
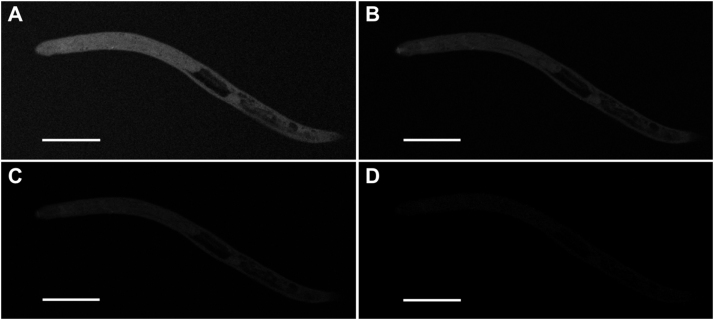


**Figure:**