# Supplementary material for: Electropermeabilization-based fluorescence in situ hybridization of whole-mount plant parasitic nematode specimens
Source: MethodsX. 2019 Nov 13;6:2720–8. doi: 10.1016/j.mex.2019.11.009 (PMC6881680; doi:10.1016/j.mex.2019.11.009)
Supplement: Supplementary file 1 [file mmc1.docx]

**Supplemental Figure 1.** **Autofluorescence of SCN pre-parasitic second-stage juvenile (ppJ2) with 405, 488, 561, and 633 nm laser lines.** **A**: 405 nm laser channel (1.5% intensity) [filter set from 449 to 498 nm], **B**: 488 nm laser channel (2.4% intensity) [filter set from 499 to 561 nm], **C**: 561 nm laser channel (2.2% intensity) [filter set from 570 to 632 nm], **D**: 633 nm laser channel (12% intensity) [filter set from 634 to 735 nm]. All channels artificially colored white within Zeiss Zen software. Scale bar represents 50 µm (Zeiss LSM 880).

**Supplemental Figure 2. Autofluorescence of SCN eggs with 561 and 633 nm laser lines. A:** 561 nm laser channel (1.5% intensity) [filter set from 570 to 632 nm], **B:** 633 nm laser (5.5% intensity) [filter set from 634 to 735 nm]. Both channels artificially colored white within Zeiss Zen software. Scale bar represents 50 µm (Zeiss LSM 880).
